# Effect of Carnosine in Experimental Arthritis and on Primary Culture Chondrocytes

**DOI:** 10.1155/2016/8470589

**Published:** 2016-01-17

**Authors:** S. Ponist, F. Drafi, V. Kuncirova, D. Mihalova, L. Rackova, L. Danisovic, O. Ondrejickova, I. Tumova, O. Trunova, T. Fedorova, K. Bauerova

**Affiliations:** ^1^Institute of Experimental Pharmacology and Toxicology, Slovak Academy of Sciences, Dubravska cesta 9, 841 04 Bratislava, Slovakia; ^2^Faculty of Medicine, Comenius University in Bratislava, Spitalska 24, 813 72 Bratislava, Slovakia; ^3^Faculty of Pharmacy, Comenius University in Bratislava, Odbojarov 10, 832 32 Bratislava, Slovakia; ^4^Research Center of Neurology, RAMS, Volokolamskoyeshosse 80, Moscow 125367, Russia

## Abstract

Carnosine's (CARN) anti-inflammatory potential in autoimmune diseases has been but scarcely investigated as yet. The aim of this study was to evaluate the therapeutic potential of CARN in rat adjuvant arthritis, in the model of carrageenan induced hind paw edema (CARA), and also in primary culture of chondrocytes under H_2_O_2_ injury. The experiments were done on healthy animals, arthritic animals, and arthritic animals with oral administration of CARN in a daily dose of 150 mg/kg b.w. during 28 days as well as animals with CARA treated by a single administration of CARN in the same dose. CARN beneficially affected hind paw volume and changes in body weight on day 14 and reduced hind paw swelling in CARA. Markers of oxidative stress in plasma and brain (malondialdehyde, 4-hydroxynonenal, protein carbonyls, and lag time of lipid peroxidation) and also activity of gamma-glutamyltransferase were significantly corrected by CARN. CARN also reduced IL-1alpha in plasma. Suppression of intracellular oxidant levels was also observed in chondrocytes pretreated with CARN. Our results obtained on two animal models showed that CARN has systemic anti-inflammatory activity and protected rat brain and chondrocytes from oxidative stress. This finding suggests that CARN might be beneficial for treatment of arthritic diseases.

## 1. Introduction

Carnosine (CARN) is dipeptide consisting of *β*-alanine and L-histidine. It was shown to be a specific constituent of excitable tissues of all vertebrates accumulating in amounts exceeding that of ATP [1]. The antioxidant capacity of this compound is well documented, as well as its pH buffering, osmoregulating, and metal-chelating abilities [2]. However, a more recent study showed unexpectedly lower binding constant values of carnosine and therefore a relatively minor role of the transition metal ion chelation in its antioxidant abilities [3]. We previously showed inhibitory properties of CARN against degradation of hyaluronan solutions at experimental conditions* in vitro*. In the reaction system with the prevalence of hydroxyl and/or peroxy-type radicals, CARN in 200 *μ*mol/L concentration tested exerted a protective action on hyaluronan degradation [4]. CARN, when compared with ascorbate, was more effective in inhibition of lipid peroxidation in meat [5]. At physiological concentrations, CARN was found to directly react with superoxide anion comparably to superoxide dismutase and the constant for interaction of carnosine with O_2_^∙−^ was calculated to be 10^5^ M^−1^·s^−1^ and not significantly different in respect to that of ascorbic acid and *α*-tocopherol [6]. Also, a sparing or regenerating effect of carnosine towards endogenous antioxidants was demonstrated in the liver of rats treated with carnosine or L-histidine. Both compounds increased the liver content of glutathione and *α*-tocopherol [7]. CARN seems to be similarly effective endogenous antioxidant as ascorbate and *α*-tocopherol. A potentially useful characteristic of CARN is its ability to act as an antiglycating agent [8–10], to quench superoxide anion and hydroxide radical [11–13], and to neutralize 4-hydroxy-nonenal (HNE) and other toxic aldehydes [14–16]. CARN may be pluripotent with respect to its ability to suppress proteotoxic stress and aging-associated phenomena [17]. CARN may suppress glycolysis, similar to the effects of rapamycin, by inhibiting TOR activity which reduces glycolytic flux, thereby decreasing the potential for methylglyoxal generation [18]. It is possible that carnosine can promote catabolism of altered proteins. Preliminary evidence was obtained suggesting that carnosine stimulates catabolism of slowly turning over proteins in aged human fibroblasts following growth for many generations with the dipeptide [19]. Oxygen metabolism has an important role in the pathogenesis of rheumatoid arthritis (RA). Reactive oxygen species (ROS) produced in the excessive amounts under some pathological states exceed the physiological ROS buffering capacity and result in oxidative stress (OS). Excessive production of ROS can damage proteins, lipids, nucleic acids, and matrix components [20, 21]. OS and decreased antioxidant status are present in patients with RA, as observed in recent years [22].

The aim of this study was to evaluate whether administration of CARN in carrageenan induced hind paw edema (model of local acute inflammatory reaction) and in adjuvant arthritis (subchronic model of rodent polyarthritis) would ameliorate inflammation, OS, and disease progression. Furthermore, in order to elucidate a potential contribution of antioxidant mechanism to these effects at cellular level,* in vitro* evaluation of the efficacy of CARN to reduce OS markers of rat primary chondrocytes exposed to hydrogen peroxide was performed, along with the assessment of their viability protection.

## 2. Methods

### 2.1. Animals, Experimental Design, and Treatments

Male Lewis rats, weighing 160–180 g, were obtained from the Breeding Farm Dobra Voda (Slovakia). The rats had free access to standard pellet diet and tap water. The animal facilities comply with the European Convention for the Protection of Vertebrate Animals Used for Experimental and Other Purposes. The experimental protocol was approved by the Ethics Committee of the Institute of Experimental Pharmacology and Toxicology and by the Slovak State Veterinary Committee of Animal Experimentation. AA was induced by a single intradermal injection of heat-inactivated* Mycobacterium butyricum* (MB) in incomplete Freund's adjuvant (Difco Laboratories, Detroit, MI, USA). The injection was performed near the tail base. The experiments included healthy animals (CO), arthritic animals (AA) not treated, and arthritic animals given 150 mg/kg b.w. of CARN daily for 28 days using gastric gavages (AA-CARN). In the acute model of inflammation, hind paw edema was induced by intraplantar injection of 0.1 mL of 1% water solution of carrageenan type IV (CARA) into the right hind paw of Lewis rats. The experiment included animals with carrageenan induced hind paw edema without any drug administration (CARA) and animals with carrageenan induced hind paw edema given carnosine (CARA-CARN). CARN was given one hour before induction of CARA in a single oral dose of 150 mg/kg b.w.

### 2.2. Blood and Tissue Collection

After the animals had been sacrificed under deep ketamine/xylazine anesthesia, blood for plasma preparation and tissues for brain, spleen, and hind paw joint homogenate preparation were taken on day 28. Heparinized plasma and tissue were stored at −70°C until biochemical and immunological analysis.

### 2.3. Clinical Parameters: Hind Paw Volume and Body Mass

Hind paw volume (HPV) increase was calculated as the percentage increase in HPV on a given experimental day relative to the HPV at the beginning of the experiment. HPV was recorded on days 1, 14, and 28 with the use of an electronic water plethysmometer (UGO BASILE). Change of body mass—CBM (g)—was measured on days 1, 14, and 28. We monitored the HPV in the CARA model at minutes 0, 30, 60, 90, 120, and 240, using the same water plethysmometer.

### 2.4. Tissue Activity of Cellular *γ*-Glutamyltransferase in Joint and Spleen Tissue

The activity of cellular *γ*-glutamyltransferase (GGT) in hind paw joint and spleen tissue homogenates was measured by the method of Orlowski and Meister [23] as modified by Ondrejickova et al. [24]. Samples were homogenized in a buffer (2.6 mM NaH_2_PO_4_, 50 mM Na_2_HPO_4_, 15 mM EDTA, and 68 mM NaCl; pH 8.1) at 1 : 9 (w/v) by UltraTurax TP 18/10 (Janke & Kunkel, Germany) for 1 min at 0°C. Substrates (8.7 mM *γ*-glutamyl-p-nitroanilide, 44 mM methionine) were added to 65% isopropylalcohol to final concentrations of 2.5 mM and 12.6 mM, respectively. After incubation for 60 min at 37°C, the reaction was stopped with 2.3 mL cold methanol and the tubes were centrifuged for 20 min at 5000 rpm. Absorbance of supernatant was measured in a Specord 40 spectrophotometer in a 0.5 cm cuvette at 406 nm. Reaction mixtures in the absence of either substrate or acceptor were used as reference samples.

### 2.5. Malondialdehyde and 4-Hydroxynonenal in Plasma and Brain Homogenates

*(1) Malondialdehyde*. ELISA (Enzyme Linked Immunosorbent Assay) Kit (Cusabio catalogue number CSB-E08557h) for the quantitative determination of endogenic MDA concentrations in plasma and tissue homogenates was used. Procedure was done according to manufacturer instructions.

*(2) 4-Hydroxynonenal*. ELISA Kit (Cusabio catalogue number CSB-E16214h) for the quantitative determination of endogenic 4-hydroxynonenal (HNE) concentrations in plasma and tissue homogenates was used. Procedure was done according to manufacturer instructions.

### 2.6. Lag Time of Fe^2+^-Induced Chemiluminescence of Plasma and Brain Homogenate

Lag time of Fe^2+^-induced chemiluminescence (LTC) of plasma was analyzed using the signal derived from addition of ferrous ions to plasma. After addition of 100 *μ*L of 25 mM FeSO_4_ to the plasma sample, the lag period between initial fast flash and the following slow rising chemiluminescence signal reflecting the rate of lipid oxidation was measured. The lag time is referring to the stability of the sample to the Fe^2+^-induced oxidation (the longer the lag period, the more stable the resistance of the biological material to oxidation) being dependent on intrinsic antioxidant capacity of plasma and brain homogenate. Chemiluminescence signal was monitored using LKB 1251 Chemiluminometer [25].

### 2.7. Protein Carbonyls in Plasma and Brain Homogenate

#### 2.7.1. Blood Plasma

ELISA was used for quantitative determination of protein carbonyls in plasma [26]. Protein samples were derivatized with dinitrophenylhydrazine (DNPH) and adsorbed in multiwell plates (NuncImmunosorp plates, Roskilde, Denmark). A biotin-conjugated anti-dinitrophenyl rabbit IgG (Sigma, USA) was used as the primary antibody and a peroxidase conjugated monoclonal anti-rabbit-IgG antibody (Sigma, USA) as the secondary antibody. The development was performed with orthophenylenediamine.

#### 2.7.2. Brain Tissue Homogenates

The amount of 250 *μ*L of 10% homogenate was added into three 2 mL test tubes for tissue blank and two parallel measurements of the sample. A 20% solution of trichloroacetic acid was added to each tube. The solutions were centrifuged for 10 min at 14 500 rpm. 200 *μ*L of 0.2 M NaOH was added into each tube. After complete dissolution of pellets, a volume of 200 *μ*L of 2 M HCl was added into each tube. 200 *μ*L of 10 mM dinitrophenyl-hydrazine solution was added only to sample tubes and incubated for 30 min. The samples were centrifuged for 10 min at 14 500 rpm. After centrifugation, the samples were washed four times with 700 *μ*L of a cooled solution of 98% ethanol : ethyl acetate (1 : 1). To each tube, 250 *μ*L of 6 M guanidine solution was added. Pellets were left to dissolve in guanidine overnight at 4°C. The concentration of proteins in each tube was measured at 280 nm and protein carbonyls were determined at 369 nm.

### 2.8. Glutathione Reductase in Brain Homogenate

The hemisphere of brain was homogenized (5% w/v) in 0.1 M sodium phosphate buffer (pH 7.0) and following centrifugation (14 000 rpm for 30 min) the supernatant was used for glutathione reductase (GR) analysis. GR activity was determined using a modification of the method described by Barker et al. [27]. The reaction mixture contained 200 *μ*L 0.1 M sodium phosphate buffer (pH 7.0), 30 *μ*L 0.1 mM NADPH, and 60 *μ*L of 5% homogenate. The reaction was initiated by the addition of 10 *μ*L GSSG (110 mg/1 mL). The oxidation of NADPH was followed at 340 nm. The molar extinction coefficient of 6.27 × 10^3^ M/cm was used to determine GR activity, and one unit of activity was defined as the number of *μ*M of NADPH oxidized min^−1^·mg^−1^ proteins.

### 2.9. Proinflammatory Cytokine IL-1*α* in Plasma

For determination of IL-1*α* concentration in plasma, ELISA Kit from R&D Systems Quantikine for IL-1 was used. Assay procedure was performed as described in the product manual. The results were calculated from standard calibration curve on internal standards.

### 2.10. Primary Chondrocytes

Primary chondrocytes were isolated from the normal articular cartilage of Wistar rats by collagenase II (0.1%) and trypsin (0.25%) digest and cultured in DMEM/Ham's F-12 supplemented with 1 mmol/L glutamine, 100 U/mL penicillin, 100 *μ*g/mL streptomycin, and 10% fetal bovine serum in CO_2_ incubator at 37°C in a humidified atmosphere containing 5% CO_2_. The culture medium was refreshed every 48 h. When cells reached the confluence, they were detached by 0.05% trypsin and subcultured up to the third passage. Chondrocytes from the 2nd and the 3rd passages were used for the experiments.

### 2.11. MTT (3-(4,5-Dimethylthiazol-2-yl)-2,5-diphenyltetrazolium Bromide) Assay

Rat primary chondrocytes were grown on 96-well microplates until confluency in 200 *μ*L DMEM/Ham's F-12. The MTT colorimetric assay was performed as previously described [28]. Briefly, the cells were preincubated for 24 hours with or without different concentrations of CARN followed by the incubation with H_2_O_2_ in DMEM/Ham's F-12 for 60 min at 37°C. MTT was added to the final concentration of 0.5 mg/mL. For the compound cytotoxicity assays, MTT was added to the wells directly following the 24-hour incubation with the substance tested. After 4 hrs, 100 *μ*L of 10% sodium dodecyl sulfate in HCl (0.01 mol/L) was added and the cells were thoroughly resuspended. The absorbance was spectrophotometrically recorded at 570 nm on Tecan Infinite 200 instrument.

### 2.12. Cell Viability Evaluation

Rat primary chondrocytes were grown on 96-well microplates until confluency in 200 *μ*L DMEM/Ham's F-12. The MTT colorimetric assay was performed as described above. The assessment of viability of primary chondrocytes was completed by fluorescence microscopy technique. The cells were grown in 96-well plates. Following incubation with H_2_O_2_, a medium was replaced by fresh medium for 5 hrs. Ethidium bromide (EB) and acridine orange dye mix (3 *μ*L) was added to each well and cells were viewed under the XDS-2 inverted fluorescence microscope [29]. Each image was collected with excitation at 488 nm. Calculations were done minimum in quadruplicate, counting a minimum of 100 total cells each.

### 2.13. Production of Intracellular Oxidants

Cellular oxidant production was determined by using dichlorodihydrofluorescein-diacetate (DCFH-DA) with a modified method as described by Giardina and Sait Inan [30]. The cells were grown in 96-well plates until confluency and then preincubated for 24 hours with CARN. Chondrocytes were then preincubated with solution of DCFH-DA (15 *μ*mol/L) for 30 min in Krebs-Ringer buffer (KRB; 10 mmol/L HEPES, 2 g/L BSA (bovine serum albumin), and pH 7.4). After the probe loading, the cells were washed with KRB and incubated with H_2_O_2_. The fluorescence of the generated dichlorofluorescein was measured on Tecan Infinite 200 instrument at an excitation wavelength of 485 nm and an emission wavelength of 528 nm.

### 2.14. Statistical Analysis 

#### 2.14.1. Cell Cultures and Relevant Assays

Each experiment was performed at least three times. Results are expressed as median. For cell culture experiments, Levene's test of equality of variances between compared groups was applied. Since the groups showed equal variances, the means were compared using unpaired Student's *t*-test. The untreated cells were compared with H_2_O_2_ exposed cells without addition of CARN (*∗*), CARN treated cells were compared with H_2_O_2_ exposed cells (+), and statistical significance was expressed as extremely significant (*p* < 0.001), highly significant (*p* < 0.01), significant (*p* < 0.05), and not significant (*p* > 0.05).

#### 2.14.2. Measurements from In Vivo Experiments

Mean and S.E.M. values were calculated for each parameter in each group (8–10 animals in each experimental group). Data in figures are expressed as mean ± S.E.M. The untreated arthritis group was compared with healthy control animals (*∗*); treated arthritis groups were compared with untreated arthritic animals (+). Statistically significant differences among treated group, untreated group, and control groups were tested using parametric Analysis of Variance (ANOVA). Alternatively, nonparametric Kruskal-Wallis test (K-W) in case of nonnormal distributed data was used. Post hoc tests (Tukey-Kramer (ANOVA), Dwass-Steel-Critchlow-Fligner (K-W)) were applied in situation where differences among groups were significant at level of significance *α* = 0.05. After* post hoc* testing, the following significance designations were specified as follows: extremely significant (*p* < 0.001), highly significant (*p* < 0.01), significant (*p* < 0.05), and not significant (*p* > 0.05).

## 3. Results 

### 3.1. Adjuvant Arthritis and Carrageenan Induced Hind Paw Edema

**Figure 1:**
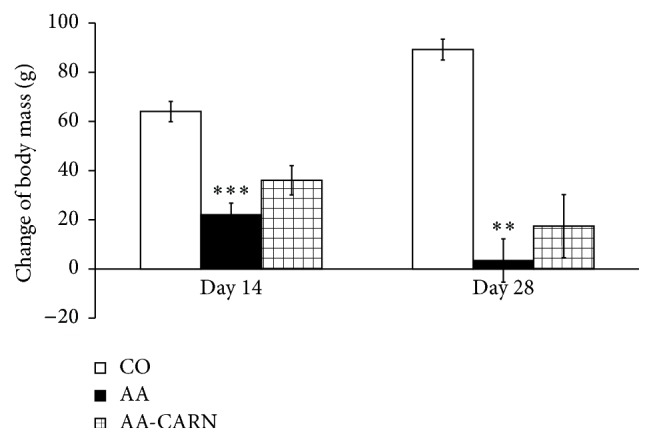
Changes in body mass of animals in adjuvant arthritis. The data are expressed as arithmetic mean with S.E.M. Each group contained 8–10 animals. ^*∗∗∗*^ *p* < 0.001 and ^*∗∗*^ *p* < 0.01 with respect to control healthy animals. The experiment included healthy intact animals as reference controls (CO), arthritic animals without any drug administration (AA), and arthritic animals with the administration of carnosine (AA-CARN).

**Figure 2:**
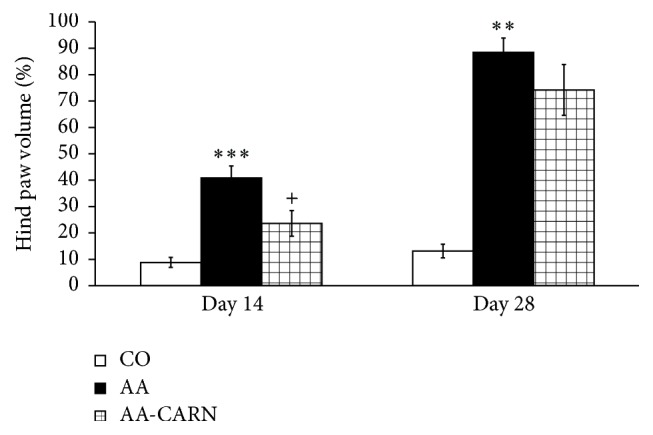
Changes in hind paw volume of animals in adjuvant arthritis. The data are expressed as arithmetic mean with S.E.M. Each group contained 8–10 animals. ^*∗∗∗*^ *p* < 0.001 and ^*∗∗*^ *p* < 0.01 with respect to control healthy animals; ^+^ *p* < 0.05 with respect to untreated arthritic animals. Groups of animals are the same as in Figure 1.

**Figure 3:**
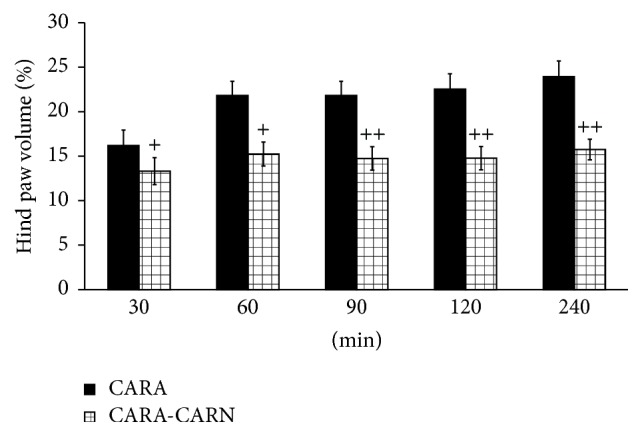
Changes in carrageenan induced edema of hind paws of Lewis rats. The data are expressed as arithmetic mean with S.E.M. Each group contained 8–10 animals. ^+^ *p* < 0.05 and ^++^ *p* < 0.01 with respect to untreated animals with carrageenan induced hind paw edema. The experiment included animals with carrageenan induced hind paw edema without any drug administration (CARA), and animals with carrageenan induced hind paw edema with the administration of carnosine (CARA-CARN).

**Figure 4:**
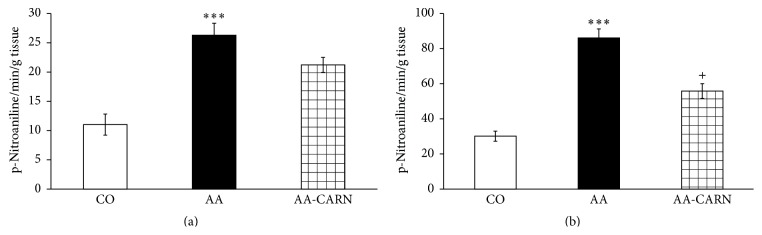
Activity of GGT in joint (a) and spleen (b) tissue homogenates in the adjuvant arthritis. The data are expressed as arithmetic mean with S.E.M. Each group contained 8–10 animals. ^*∗∗∗*^ *p* < 0.001 with respect to control healthy animals; ^+^ *p* < 0.05 with respect to untreated arthritic animals. Groups of animals are the same as in Figure 1.

**Figure 5:**
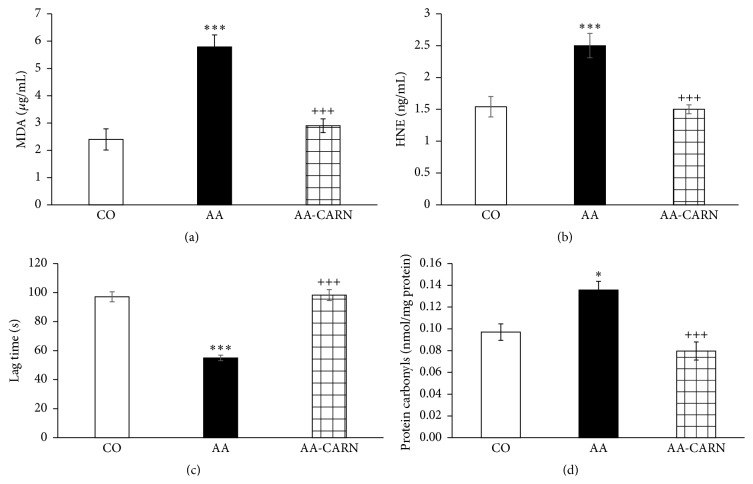
Markers of oxidative stress measured in the plasma of rats with adjuvant arthritis. Level of MDA (a) and HNE (b), lag time of Fe^2+^-induced chemiluminescence (c), and levels of protein carbonyls (d). The data are expressed as arithmetic mean with S.E.M. Each group contained 8–10 animals. ^*∗∗∗*^ *p* < 0.001 and ^*∗*^ *p* < 0.05 with respect to control healthy animals; and ^+++^ *p* < 0.001 with respect to untreated arthritic animals. Groups of animals are the same as in Figure 1.

**Figure 6:**
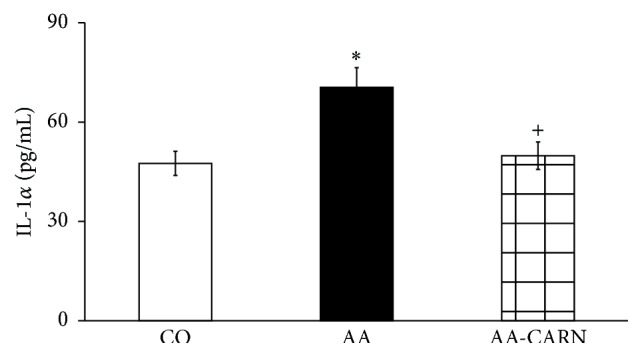
Level of IL-1*α* in plasma in the adjuvant arthritis. The data are expressed as arithmetic mean with S.E.M. Each group contained 8–10 animals. ^*∗*^ *p* < 0.05 with respect to control healthy animals; ^+^ *p* < 0.05 with respect to untreated arthritic animals. Groups of animals are the same as in Figure 1.

**Figure 7:**
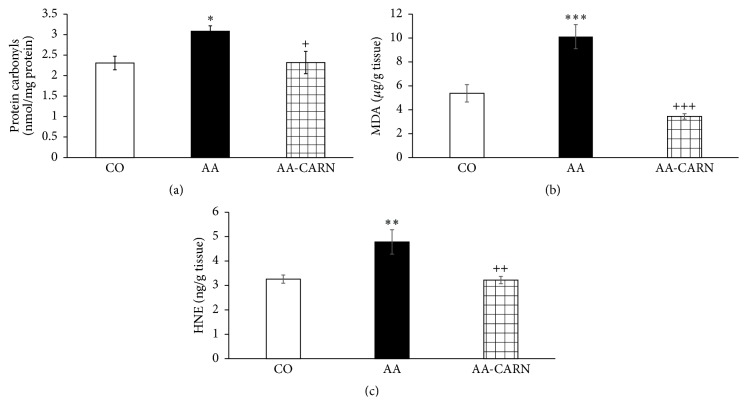
Level of protein carbonyls (a), MDA (b), and HNE (c) in brain tissue of rats with adjuvant arthritis. The data are expressed as arithmetic mean with S.E.M. Each group contained 8–10 animals. ^*∗*^ *p* < 0.05, ^*∗∗*^ *p* < 0.01, and ^*∗∗∗*^ *p* < 0.001 with respect to control healthy animals; ^+^ *p* < 0.05, ^++^ *p* < 0.01, and ^+++^ *p* < 0.001 with respect to untreated arthritic animals. Groups of animals are the same as in Figure 1.

**Figure 8:**
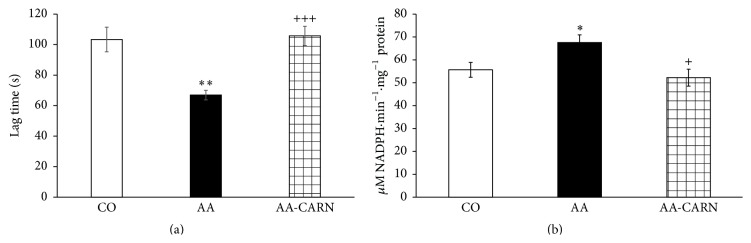
Lag time of chemiluminescence (a) and activity of glutathione reductase (b) in brain tissue of rats with adjuvant arthritis. The data are expressed as arithmetic mean with S.E.M. Each group contained 8–10 animals. ^*∗*^ *p* < 0.05, ^*∗∗*^ *p* < 0.01 with respect to control healthy animals; ^+^ *p* < 0.05, ^+++^ *p* < 0.001 with respect to untreated arthritic animals. Groups of animals are the same as in Figure 1.

CARN beneficially affected clinical parameters (change of body mass and hind paw volume) in the model of AA measured on days 14 and 28 (Figures 1 and 2) and hind paw volume significantly at day 14 (Figure 2). CARN also reduced hind paw volume in the model of CARA during the whole experiment: 30 min–240 min (Figure 3), most effectively at 240 min. Reduction of hind paw volume by CARN treatment in AA on day 14 was 42% and in CARA model 34% at 240 min, when compared to untreated arthritic animals. Activity of GGT in joint and spleen homogenates from arthritic animals was reduced by CARN administration (Figures 4(a) and 4(b)) significantly in spleen tissue—35% (Figure 4(b)). CARN decreased lipid peroxidation in plasma assessed as MDA and HNE (Figures 5(a) and 5(b)). Moreover, plasmatic proteins were protected against oxidation occurring in AA development very effectively by CARN administration (Figure 5(d)). Also total resistance of plasma against Fe^2+^-induced oxidation was significantly increased by CARN measured as lag time of chemiluminescence (Figure 5(c)). AA increased the levels of inflammation marker IL-1*α* in plasma (Figure 6). Our results point out to connection between OS and immune response in AA, because the decrease of OS markers (MDA, HNE, LTC, and protein carbonyls) was simultaneously accompanied by reduction of immunological marker IL-1*α* in plasma by CARN. This result is showing that CARN can have also anti-inflammatory activity (Figure 6). AA caused increased oxidative stress in brain tissue measured by MDA, HNE, and protein carbonyls. CARN completely prevented the damage done by oxidative stress to lipids and proteins (Figures 7(a), 7(b), and 7(c)). Lowered antioxidant capacity of brain tissue was measured as decreased lag time of chemiluminescence and was corrected by CARN administration (Figure 8(a)). Activity of GR in brain tissue homogenates of arthritic animals was significantly elevated and was decreased by CARN to control values (Figure 8(b)).

### 3.2. Primary Cell Culture of Chondrocytes

**Figure 9:**
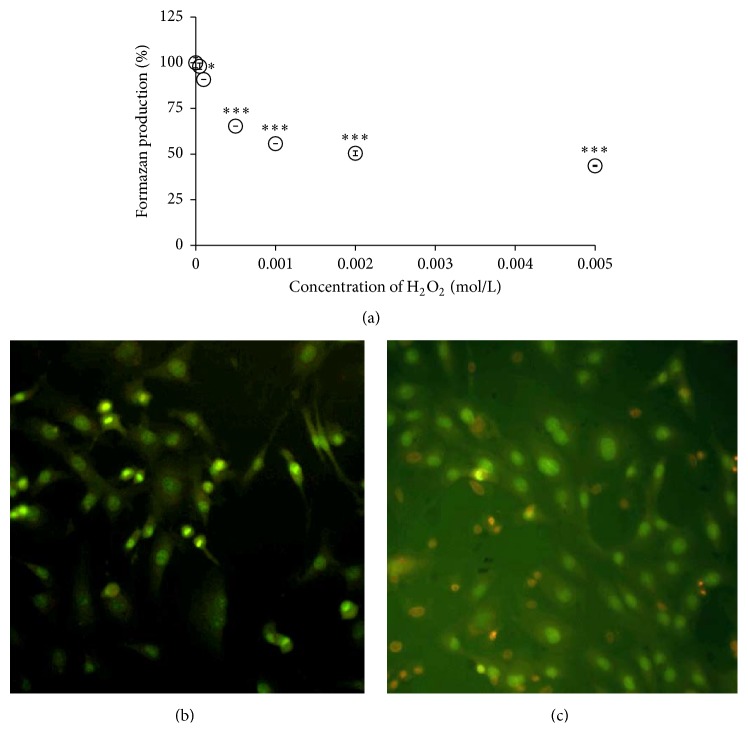
Vitality changes of primary rat chondrocytes incubated with hydrogen peroxide. Concentration-dependent effect of H_2_O_2_ on MTT viability (a) and combined fluorescence labeling of the cells with ethidium bromide and acridine orange (b, c). Images were captured 5 hours following incubation without (b) or with H_2_O_2_ (5 mmol/L, 400x) (c). Results are expressed as arithmetic mean with S.E.M. ^*∗*^ *p* < 0.05 and ^*∗∗∗*^ *p* < 0.001 with respect to control.

**Figure 10:**
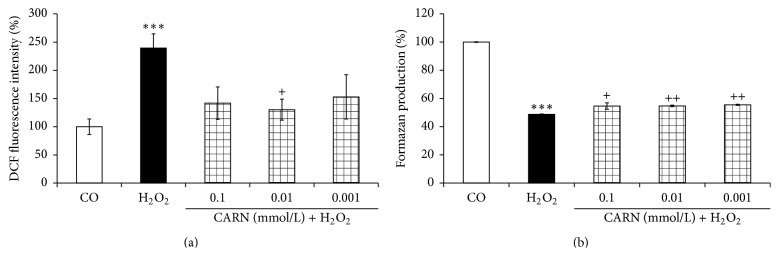
Effect of CARN on intracellular oxidant levels (a) and on viability injury (b) in rat primary chondrocytes exposed to H_2_O_2_. The cells were preincubated for 20 hours with the compound tested. Results are expressed as median. ^*∗∗∗*^ *p* < 0.001 with respect to control and ^++^ *p* < 0.01 and ^+^ *p* < 0.05 with respect to H_2_O_2_ control.

Cytotoxicity of hydrogen peroxide to primary chondrocytes showed a clear concentration-dependent profile (Figure 9(a)). The MTT viability was reduced to 43.6 ± 0.6% of control formazan production upon treatment with maximum 5 mM H_2_O_2_ (Figure 9(a)). In addition, representative image (Figure 9(c)) shows the concomitant increase of EB-positive dead cells (by 48.4 ± 4.6%) in primary chondrocytes exposed to H_2_O_2_ (5 mM) in comparison to untreated cells (0%, Figure 8(b)). An apparent lack of mitotic cells was also observed in chondrocytes exposed to H_2_O_2_ (yielding 0.9 ± 0.5%, Figure 9(c)) in contrast to a noticeable number of dividing cells in untreated control (11.2 ± 1.8%, calculated as mother cells and daughter pair cells with bright condensed nuclear chromatin, Figure 8(b)). In spite of a remarkable decrease of MTT reduction by chondrocytes treated with 1 mM H_2_O_2_ (55.7 ± 0.0%), only a negligible increase of EB-positive cells was observed at this concentration (data not shown). Pretreatment with CARN reduced considerably the intracellular levels of oxidants in 5 mM H_2_O_2_-stressed primary chondrocytes (Figure 10(a)), which was accompanied with a moderate but significant prevention of viability injury of the cells (Figure 10(b)). For the concentration range tested, no dose dependency was found.

## 4. Discussion

The reactive oxygen and nitrogen species can react with lipids, proteins, and nucleic acids and are thought to be of importance for the etiology of chronic inflammatory rheumatic diseases [20]. One approach to counteract this OS situation is the use of antioxidants as therapeutic agents. CARN was found to have neuroprotective, hepatoprotective, and antiaging abilities [31] as well as antiradical activity [12, 13]. Nevertheless, its anti-inflammatory potential in autoimmune systemic inflammatory diseases, as RA, has been scarcely investigated as yet.

The GGT activity was elevated in peripheral joint and spleen tissue. CARN effectively reduced the activity of GGT in spleen homogenates and slightly in joint. In our previous studies with coenzyme Q_10_ [32] and glucomannan [33], the reduction of activity of GGT in spleen was accompanied also by beneficial improvement of clinical markers of AA disease. Basaran-Küçükgergin et al. [34] showed the ability of CARN to decrease the GGT activity in serum of diethylnitrosamine-induced OS and tissue injury in liver of rats. RA was associated with significant depletion (ca. 50%) in glutathione levels compared with normal control subjects. Serum levels of the detoxifying enzymes GR and glutathione peroxidase decreased by ca. 50% and 45%, respectively. These results support a hypothesis that defense mechanisms against reactive oxygen species are impaired in RA [35–37]. In our experiment, we found increased activity of GR in brain tissue of arthritic animals, which was decreased to basal values after administration of CARN. This result is suggesting a higher turnover of glutathione in brain during AA, probably due to increased OS also in this tissue (see below the increased protein carbonyl level, MDA, HNE, and LTC in brain tissue). Unfortunately, there is a lack of studies about CARN affecting GR; thus the mechanism how CARN decreases GR activity remains unclear. CARN increased the LTC in plasma samples, which refers to its ability to restore the systemic antioxidant capacity of plasma. CARN has shown a good protective activity against LTC as human plasma lipoproteins* in vitro* and brain of experimental animals [38]. In the present experiment, we report for the first time on the protective effect of CARN on plasmatic LTC and on GR activity in brain of rats with AA. In animal models of AA, the level of MDA was elevated in the plasma [39, 40]. Administration of CARN lowered the level of secondary products of lipid peroxidation in plasma measured as MDA and HNE. There is only little information about OS and brain damage in the literature [41, 42]. For the first time, we evidenced increased HNE and reduced lag time of chemiluminescence in rat brain during AA. Although RA is not a typical CNS involvement disease, brain dysfunctions occur in 20 to 30% of rheumatic patients [43]. In the hippocampus of AA animals, upregulation of mRNA for IL-1*β*, IL-6, and markers of oxidative stress-inducible NO synthase and NADPH oxidase-1 were observed within four days. The changes correlated with anxiety-like behavior [42]. Elevated levels of protein carbonyls were found in experimental animals [44]. In this paper, we report for the first time a significant elevation of protein carbonyls in brain of rats with AA. This novel finding emphasizes the systemic role of OS in chronic inflammatory diseases such as AA with oxidatively modified proteins not in directly affected tissues only (cartilage, bone, and skeletal muscle). Other authors also found CARN to reduce protein carbonyls. CARN decreased protein carbonyl levels in both liver and brain tissues in several tissues of rats exposed to chronic cold plus immobilization stress [45]. In our experiment, CARN showed complex protective effects against OS induced depletion of plasma antioxidants assessed by LTC, lipid peroxidation measured as MDA and HNE, and protein carbonylation.

The outcome of our* in vitro* experiments shed light partially also on an alternative mechanism of antioxidant action of CARN. Hydrogen peroxide, a highly reactive substance involved in the pathogenesis of RA, caused a concentration-dependent viability decrease of primary chondrocytes (principal components of articular cartilage). Thus CARN may provide also prevention of oxidative damage and tissue injury in RA development. We did not find typical dose-dependent effect for the concentration range tested, suggesting indirect mechanism of protection by CARN against free radical damage derived from H_2_O_2_ likely linked to its referred hormetic properties [46]. In agreement with works reporting on a biphasic dose-response curve for the substances inducing hormesis [47], we found the maximum suppression of ROS levels in the cells pretreated with the middle concentration tested (10 *μ*M) of CARN.

The action of CARN resulted in decreased systemic inflammation in AA, monitored by plasmatic level of proinflammatory cytokine IL-1*α*. Recent research has shown that in the processes of RA IL-1 is one of the pivotal cytokines in initiating the disease. In patients with RA and related spondyloarthropathies, IL-1 and TNF*α* are key contributors [48, 49]. In AA, CARN significantly reduced the level of IL-1*α* in plasma, but this effect resulted only in mild reduction of HPV on day 28. However, while inhibition of IL-1, TNF*α*, or both yields a significant anti-inflammatory effect in rats with AA, residual disease persists [32]. Beneficial effects of CARN manifested in reduction of systemic OS and reduced level of IL-1*α* in plasma were accompanied also by reduction of HPV and CBM. CARN beneficially affected HPV and CBM measured on day 14 and on day 28, significantly on day 14 when the clinical manifestation of the disease started. CARN was able to delay the disease onset. CARN also reduced HPV in the model of CARA during the whole experiment, 30 min–240 min, and was more effective in this animal model of acute inflammation. One of the possible explanations of HPV reduction is that restoration of redox balance in AA could have inhibitory effects on some immune cells and cytokine signaling involved in disease.

## 5. Conclusion

We showed a protective effect of CARN against oxidative damage on chondrocytes, which may be helpful in preventing cartilage degradation in “arthritic” joint. CARN also reduced the level of protein carbonyls and the activity of glutathione reductase in the brain of animals with AA, which is a unique finding for this animal model of arthritis. Our results from two animal models indicate that CARN may have also systemic anti-inflammatory effects. However, it still remains unclear if the ability of CARN to restore redox balance is the only mechanism responsible for its anti-inflammatory effects in AA and CARA. Nevertheless, CARN administered together with standard antirheumatic therapy could enhance its effectivity and it might become a potential candidate to enrich the repertoire of anti-inflammatory drugs in the future.
